# Discovery of Novel Viruses and Common Contaminants From Unmapped DNA and RNA in Pigs and Chickens Under Different Housing Conditions

**DOI:** 10.1002/age.70121

**Published:** 2026-05-13

**Authors:** Martijn F. L. Derks, Bert Dibbits, Richard P. M. A. Crooijmans, Kim Lensing

**Affiliations:** ^1^ Animal Breeding and Genomics Wageningen University & Research Wageningen the Netherlands

**Keywords:** contaminants, livestock genomics, unmapped reads, virome

## Abstract

Unmapped sequencing reads in livestock often contain valuable information about pathogens but are typically discarded. We analyzed blood‐derived DNA and RNA from chickens and pigs kept under high‐ and low‐biosecurity conditions, focusing on unmapped reads. In chickens, low‐biosecurity farms harbored substantially more viral sequences, primarily plant viruses, indicating environmental contamination. In pigs, *Mycoplasmoides pneumoniae* and several pig‐specific viruses were detected. Our bioinformatics pipeline, involving host read removal, assembly, BLAST, and taxonomic filtering, efficiently identified candidate pathogens and contaminants. This approach demonstrates the potential of sequencing‐based environmental DNA monitoring to track microbial and viral presence, assess farm biosecurity, and support animal health surveillance.

## 1 Introduction

Resequencing is routinely performed in pig and chicken breeding programs, particularly within purebred lines, to monitor genetic diversity, track selection, and identify potential causative variants for economically important traits (Ros‐Freixedes 2024). However, during these efforts, a substantial fraction of sequencing reads often remains unmapped to the host genome. These unmapped reads are typically discarded, despite the fact that they may contain valuable information, especially regarding the presence of pathogens such as viruses, bacteria, or other microbial agents (Bovo et al. 2022). By overlooking these reads, an important layer of biological information that could influence animal health, performance, or disease resistance is potentially being missed.

In this study we investigate the relationship between farm biosecurity level and pathogen presence in blood‐derived sequencing data. We selected 10 chicken ( *Gallus gallus* ) samples from a low‐biosecurity farm and 10 from a high‐biosecurity farm for whole‐genome sequencing, focusing specifically on analysing the unmapped reads. A similar design was applied in pigs ( *Sus scrofa* ), with 5 samples from each type of farm; however, for pigs we also performed total RNA sequencing, enabling the detection of RNA viruses in addition to DNA‐based pathogens.

This approach allows us to evaluate the potential of unmapped reads as a surveillance tool for monitoring pathogen load and to assess whether housing conditions significantly influence the microbial and viral landscape in livestock populations.

## 2 Methods

We collected blood samples from chickens and pigs kept under different biosecurity conditions. In total, 20 chicken samples were obtained, comprising 10 from high‐biosecurity farms and 10 from conventional farms with lower biosecurity and likely higher pathogen load. Additionally, 10 pig samples were collected, with 5 from a high‐biosecurity farm and 5 from a conventional farm. For pigs, the high‐biosecurity farm was a Specific Pathogen Free (SPF) nucleus breeding farm with strict biosecurity measures, while the low‐biosecurity farm was a conventional commercial sow farm producing three‐way crossbred finisher pigs under standard industry conditions. For chickens, the high‐biosecurity farm was a nucleus breeding farm with controlled breeding stock and strict hygiene protocols, whereas the low‐biosecurity farm was a conventional laying hen farm with typical commercial biosecurity practices. Genomic DNA and total RNA were extracted from whole blood using standard protocols. Total RNA was extracted using the RNeasy Mini Kit (Qiagen). Library preparation was performed by Novogene, using an rRNA depletion protocol (no poly(A) enrichment). All samples were sequenced using paired‐end 150 bp reads on an Illumina HiSeq 4000. The average median insert size was 194 bp for chicken samples and 188 bp for pig samples, and no spike‐in controls were used during sequencing. All samples were subjected to whole‐genome sequencing with an average coverage of 13.7× (chicken) and 15.2 (pig) (Tables S1 and S2). In addition, total RNA sequencing was performed for the pig samples.

**FIGURE 1:**
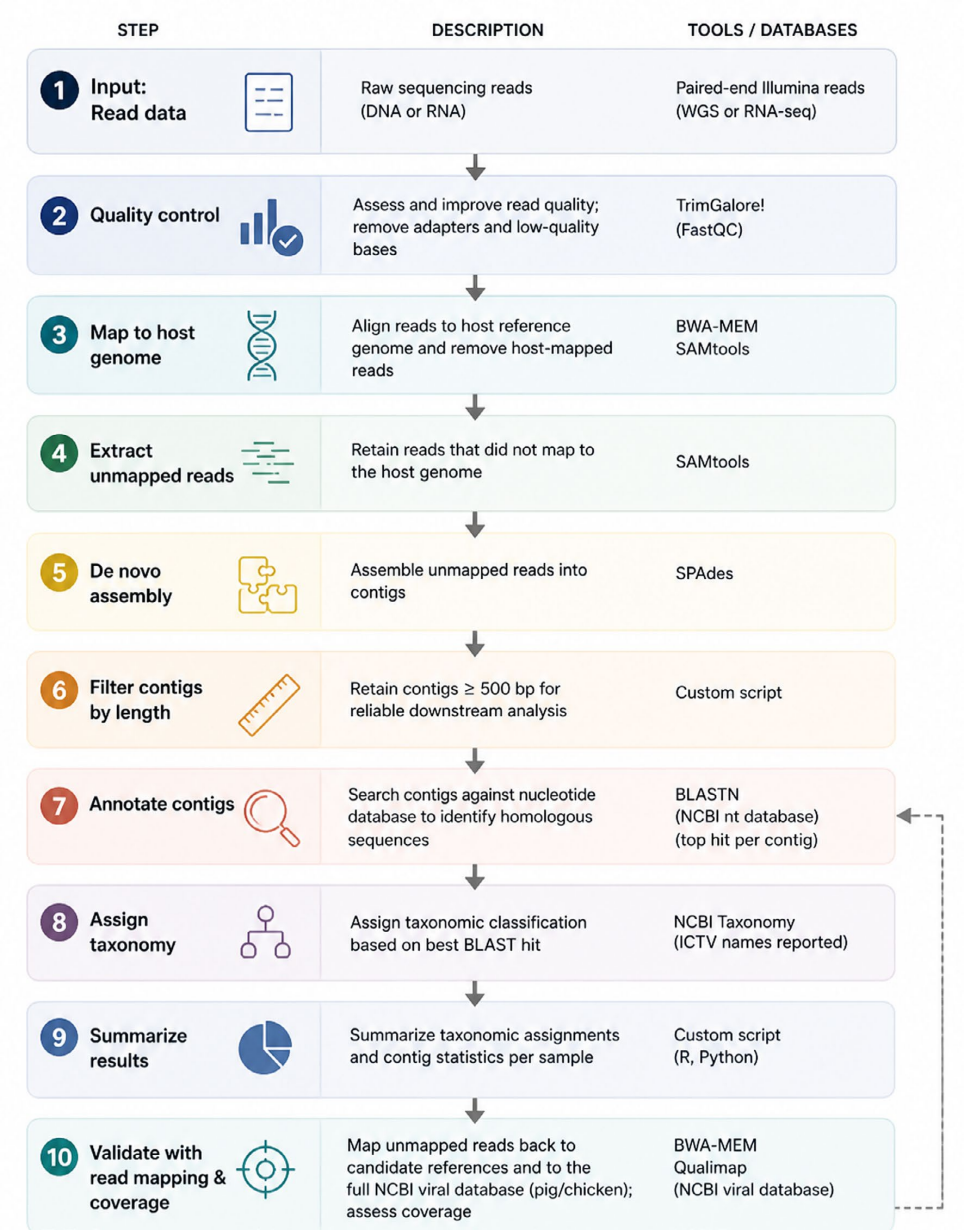
Bioinformatics pipeline to detect candidate pathogens and common contaminants in WGS and RNA‐seq data.

To extract relevant non‐host DNA and RNA, we designed a bioinformatics pipeline (Figure 1). Chicken reads were aligned to the *GRCg7b* reference genome (Smith et al. 2023) and pig reads to the T2T Landrace genome (GCA_963921485.1) using BWA‐MEM2 (Vasimuddin et al. 2019). Unmapped paired‐end reads were extracted with samtools 1.22 (Danecek et al. 2021) and subsequently reassembled using SPAdes v4.0.0 with default parameters (Prjibelski et al. 2020). Contigs shorter than 500 bp were removed, and the remaining contigs were queried against the NCBI nt database using BLAST (accessed May 2025) (Camacho et al. 2009), retaining hits with an *e*‐value < 0.01. Results were visualized in matplotlib (Hunter 2007), and further filtering by kingdom, class, family, and species was performed with a custom Python script. Candidate pathogen hits were further validated by mapping reads back to the corresponding reference genomes and the complete NCBI viral database (chicken or pig) (Brister et al. 2015) to confirm coverage and reduce false positives, this coverage was assessed with Qualimap (Okonechnikov et al. 2016). The results of the coverage analysis are in Table S3 (chicken WGS), Table S4 (pig WGS), and Table S5 (pig RNA). Analyses focused on parasitic eukaryotes, fungi, bacteria, and viruses as candidate pathogens. The same pipeline was applied to the RNA‐seq data from pigs.

## 3 Results and Discussion

In chickens, the proportion of unmapped reads ranged from 0.46% to 1.36%. Assembly of these reads yielded 1725–4610 contigs per sample (Table 1, Table S1). The majority of contigs matched avian sequences, though several sources of contamination were observed. One sample (5) contained ~1600 human‐derived contigs, another (12) contained 161 contigs matching *Epinephelus fuscoguttatus* (brown‐marbled grouper), and six samples had > 100 contigs from plant (eudicot) DNA (Figure S1). *Fusarium oxysporum* was detected in sample 19 (Figure S2), likely reflecting feed or environmental contamination. Three samples contained > 400 contigs matching *Pseudomonas fluorescens* and *Pedobacter* (Figure S3), both known DNA extraction kit contaminants (Salter et al. 2014). Microbiome‐associated species were also identified, including *Collinsella* (20 contigs, sample 20) and *Phocaeicola* (39 contigs, sample 16). Viral content differed markedly between groups: only 4 viral contigs were detected in the high‐biosecurity group, whereas 240 were identified in the low‐biosecurity group (Figure S4). Of the 15 viral taxa detected, 5 were plant viruses (e.g., *Fabavirus betaviciae*), 6 bacteriophages, 1 yeast‐infecting virus, and 1 contig resembling SARS‐CoV‐2. Most of these viruses likely represent environmental contamination, but the striking difference between high‐ and low‐biosecurity farms highlights the greater presence of exogenous viral material in low‐biosecurity conditions.

**TABLE 1:** Result summary of candidate pathogens and contaminants.

Species	Sample type	% unmapped reads	Contigs (> 500 bp)	Contaminants	Pathogens and viruses
Chicken	DNA (20 samples)	0.46–1.36	1725–4610	Human DNA; fish ( *E. fuscoguttatus* ); plant DNA; fungi (*Fusarium oxysporum*); kit contaminants (*Pseudomonas*, *Pedobacter*); microbiome taxa (*Collinsella*, *Phocaeicola*)	4 viral contigs (high biosecurity) vs. 240 (low biosecurity); mostly plant viruses, bacteriophages, one yeast virus, one SARS‐CoV‐2‐like contig
Pig	DNA (10 samples)	0.26–0.41	1384–2932	Plant DNA; fungi in 3 samples; *Pseudomonas* in all; additional *Pedobacter*, *Sphingobacterium*, *E. coli*	*Mycoplasoides pneumoniae*
Pig	RNA (10 samples)	0.6–0.9	1006–2329	Rainbow trout; *E. coli; Cutibacterium acnes*, *Shigella sonnei*	22 viral contigs, including *Porcine parvovirus*, *Torque teno sus virus*, *Porcine lymphotropic herpesvirus*

**FIGURE 2:**
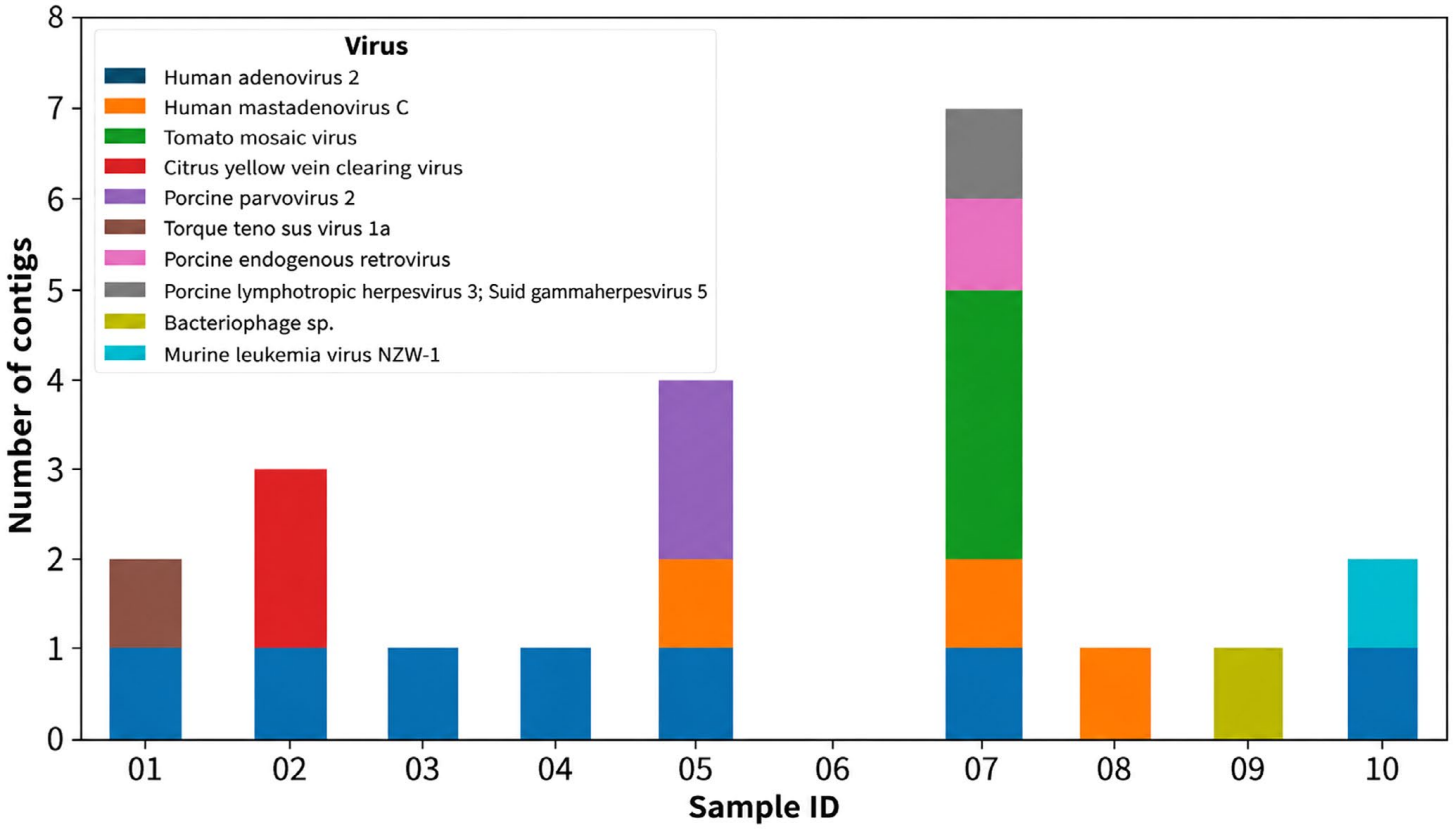
Number viral hits in pigs. Contig count is given on the y‐axis, sample numbers on the x‐axis. Sample 1–5 is from high biosecurity farm while sample 6–10 are from low biosecurity.

In pigs, unmapped reads represented 0.26%–0.41% of DNA data, yielding 1384–2932 contigs per sample. RNA sequencing yielded 1006–2329 contigs per sample (Table S2). For DNA, the majority of contigs matched pig or other even‐toed ungulates, with additional matches to plants (Figure S5). Several samples (1, 8, and 9) contained > 10 fungal contigs, mostly plant‐associated (Figure S6). All samples contained *Pseudomonas* contamination (> 50 contigs), and sample 6 had ~250 additional bacterial contigs including *Pedobacter*, *Sphingobacterium*, and *E. coli* (Figure S7). Importantly, *Mycoplasoides pneumoniae* was detected in samples 1, 8, and 10, a pathogen causing mild infectious pneumonia in pigs. No DNA viruses were detected. For RNA, the majority of contigs again mapped to pig or related species (Figure S8). Contamination with rainbow trout was observed in sample 5 (> 600 contigs), while *E. coli* was abundant in eight samples (> 50 contigs) (Figure S9), despite lacking an RNA genome, suggesting technical contamination. Sample 7 contained > 100 contigs from *Cutibacterium acnes* (human skin) and *Shigella sonnei* . A total of 22 viral contigs were detected, including pathogens like *Tetraparvovirus ungulate3 (Porcine parvovirus*) in sample 5, *Iotatorquevirus suida1a* (*Torque teno sus virus*), and *Macavirus suidgamma5* (*Porcine lymphotropic herpesvirus*) (Figure 2). No differences were observed between high‐ and low‐biosecurity groups in pigs.

In summary, our analyses reveal that chickens from low‐biosecurity farms carried substantially more viral sequences than those from high‐biosecurity farms. However, the majority of these were plant viruses, suggesting environmental contamination. A small number of animal viruses were also identified, including RNA viruses. In pigs, contamination with *Pseudomonas* was universal, and the pathogen *Mycoplasoides pneumoniae* was detected in several individuals, consistent with its role in mild pneumonia. Our analysis revealed several known porcine viruses (e.g., *Torque teno sus virus*, *Porcine parvovirus*), though notably no evidence for porcine reproductive and respiratory syndrome virus (PRRSV), a common pathogen in pig breeding. A limitation of our assembly‐based approach is that it preferentially detects more abundant organisms, and low‐abundance viral sequences may fail to assemble into sufficiently long contigs, potentially leading to an underestimation of low‐level infections.

This pipeline demonstrates the potential of sequencing‐based approaches to monitor pathogen presence in farm environments. By removing host reads and focusing on non‐host DNA and RNA, candidate pathogens from diverse taxonomic groups can be identified and tracked. Such an approach could be applied to eDNA samples collected from wastewater, dust, or other farm‐associated materials, offering a non‐invasive and cost‐effective method to survey pathogen load (Kestel et al. 2022). Routine monitoring of farms using this strategy would provide early warning of pathogen outbreaks, improve biosecurity management, and contribute to safeguarding both animal and public health.

## Funding

This work was supported by the Dutch Ministry of Agriculture, Fisheries, Food Security and Nature (LWV23008) and the Breed4Food BRIGHT (BO‐68‐001‐069).

## Conflicts of Interest

The authors declare no conflicts of interest.

## Supporting information


**Figure S1:** Number of hits with eukaryotic species in chicken WGS set.
**Figure S2:** Number of hits with fungi, mites, and ticks in chicken WGS set.
**Figure S3:** Number of hits with bacteria chicken WGS set.
**Figure S4:** Number of hits with viruses in chicken WGS set.
**Figure S5:** Number of hits with eukaryotes in pig WGS set.
**Figure S6:** Number of hits with fungi, mites, and ticks in pig WGS set.
**Figure S7:** Number of hits with bacteria in pig WGS set.
**Figure S8:** Number of hits with eukaryota in pig RNA‐seq dataset.
**Figure S9:** Number of hits with bacteria in pig RNA‐seq dataset.

**Table S1:** Mapping and assembly statistics in chicken dataset.
**Table S2:** Mapping and assembly statistics in pig WGS and RNA‐seq datasets.
**Table S3:** Coverage of unmapped WGS reads aligned to blast hit species and NCBI viral database (chicken).
**Table S4:** Coverage of unmapped WGS reads aligned to blast hit species and NCBI viral database (pigs).
**Table S5:** Coverage of unmapped total RNA reads aligned to blast hit species and NCBI viral database (pigs).

## Data Availability

Sequence data supporting this study has been submitted to ENA under BioProject: PRJEB102565. The pipeline and scripts used are available from https://git.wur.nl/kim.lensing/unmapped‐reads.
