# Supplementary material for: Discovery of Novel Viruses and Common Contaminants From Unmapped DNA and RNA in Pigs and Chickens Under Different Housing Conditions
Source: Anim Genet. 2026 May 13;57:e70121. doi: 10.1002/age.70121 (PMC13170512; doi:10.1002/age.70121)
Supplement: Supplementary file 1 — Figure S1: Number of hits with eukaryotic species in chicken WGS set. Figure S2: Number of hits with fungi, mites, and ticks in chicken WGS set. Figure S3: Number of hits with bacteria chicken WGS set. Figure S4: Number of hits with viruses in chicken WGS set. Figure S5: Number of hits with eukaryotes in pig WGS set. Figure S6: Number of hits with fungi, mites, and ticks in pig WGS set. Figure S7: Number of hits with bacteria in pig WGS set. Figure S8: Number of hits with eukaryota in pig RNA‐seq dataset. Figure S9: Number of hits with bacteria in pig RNA‐seq dataset. [file AGE-57-0-s001.docx]

**Supplementary figures**


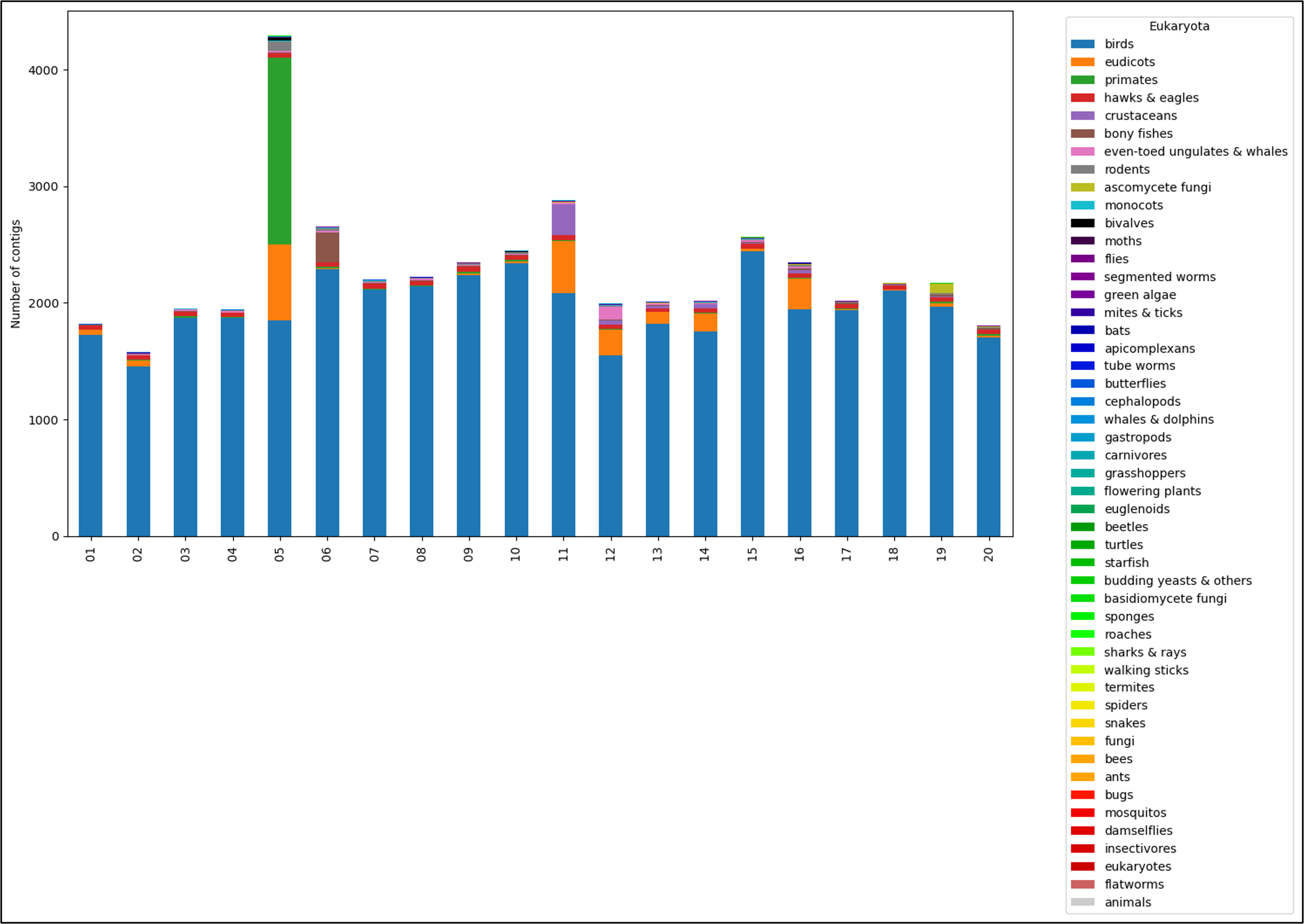


Figure S1: Number of hits with eukaryotic species in chicken WGS set. Contig count is given on the y-axis, sample numbers on the x-axis

**
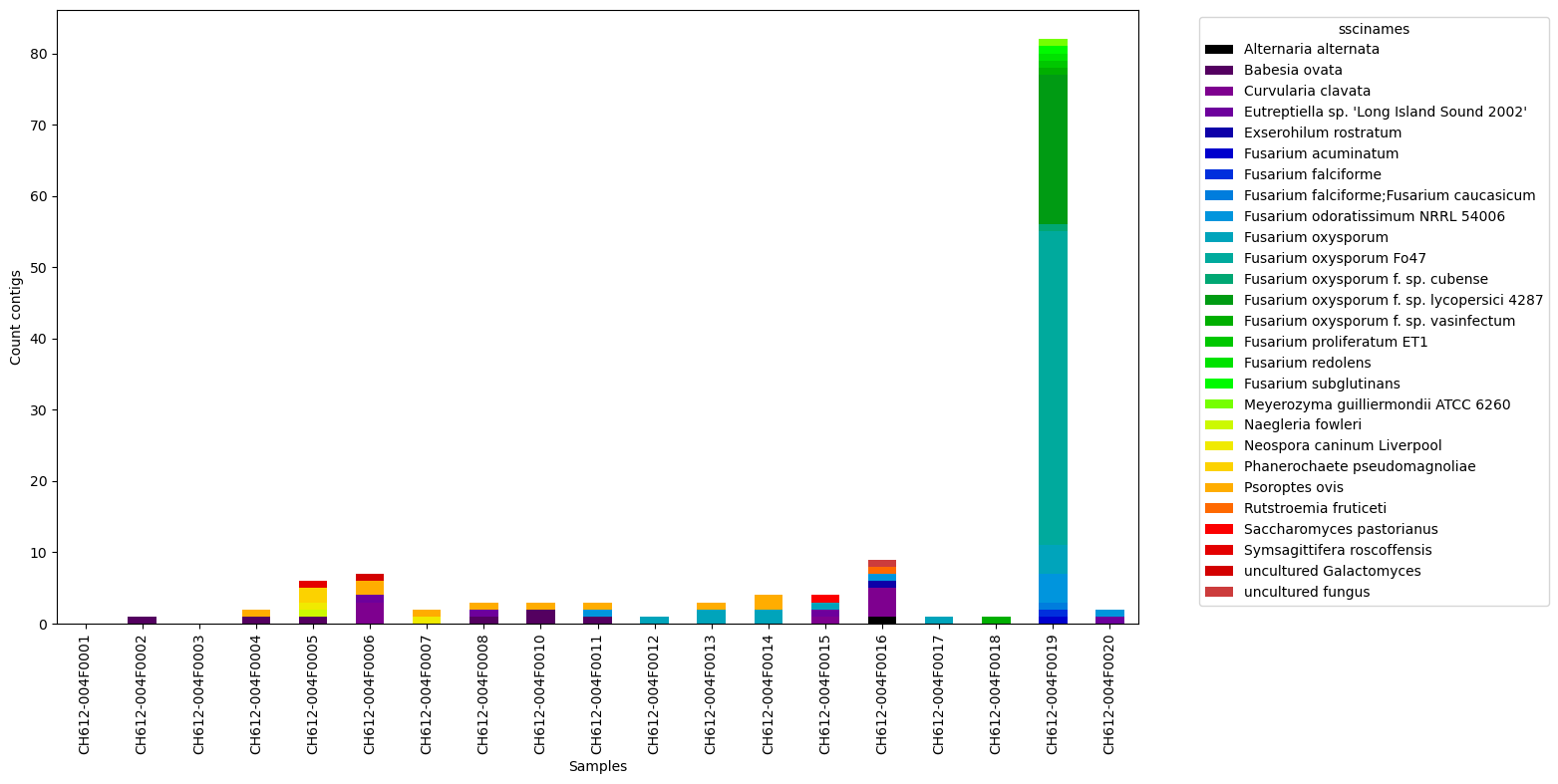
**

Figure S2: Number of hits with fungi, mites, and ticks in chicken WGS set. Contig count is given on the y-axis, sample numbers on the x-axis

**
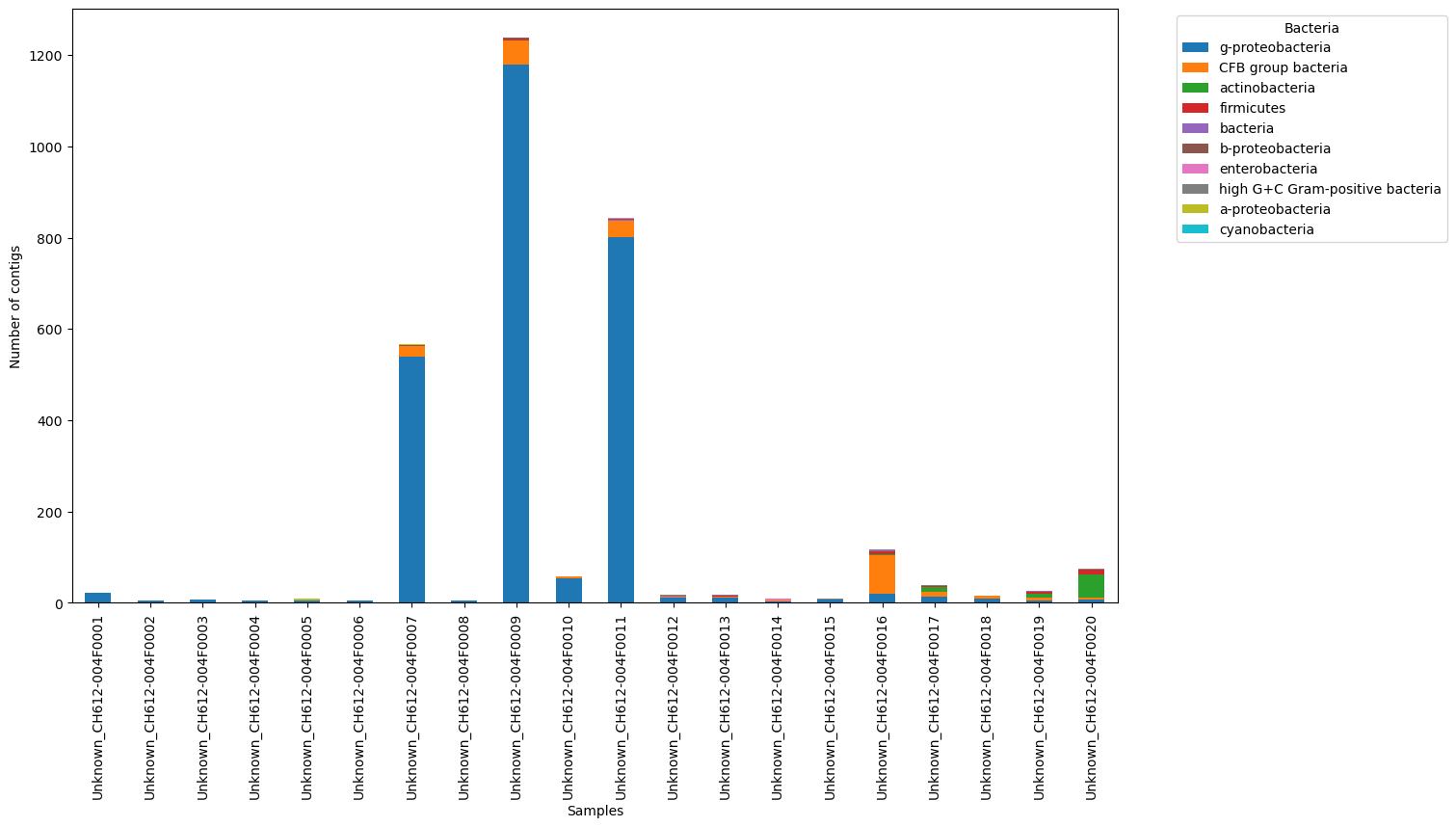
**

Figure S3: Number of hits with bacteria chicken WGS set. Contig count is given on the y-axis, sample numbers on the x-axis


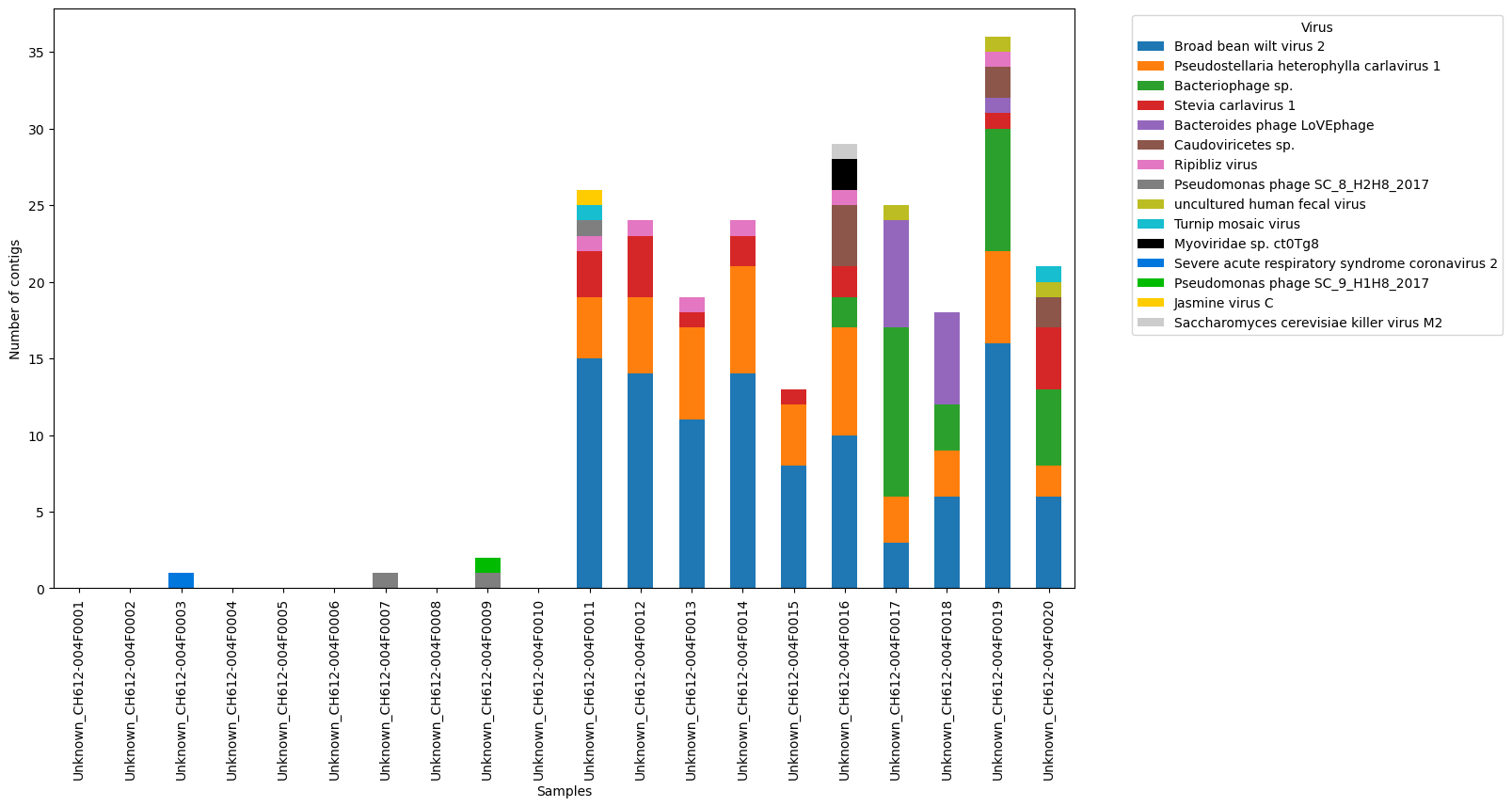

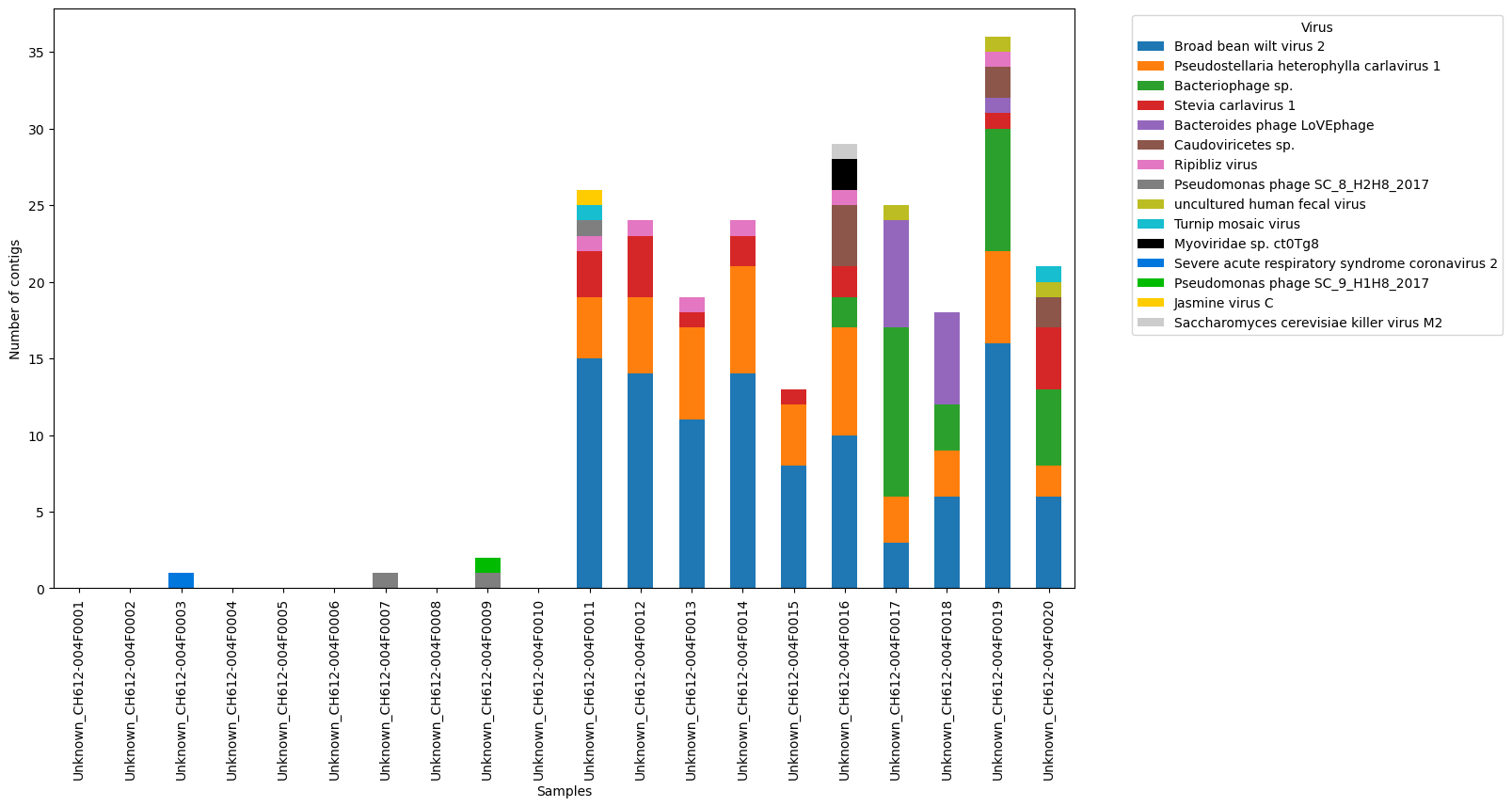


Figure S4: Number of hits with viruses in chicken WGS set. Contig count is given on the y-axis, sample numbers on the x-axis. Sample 1-10 are from a high biosecurity farm and sample 11-20 are from a low biosecurity farm.


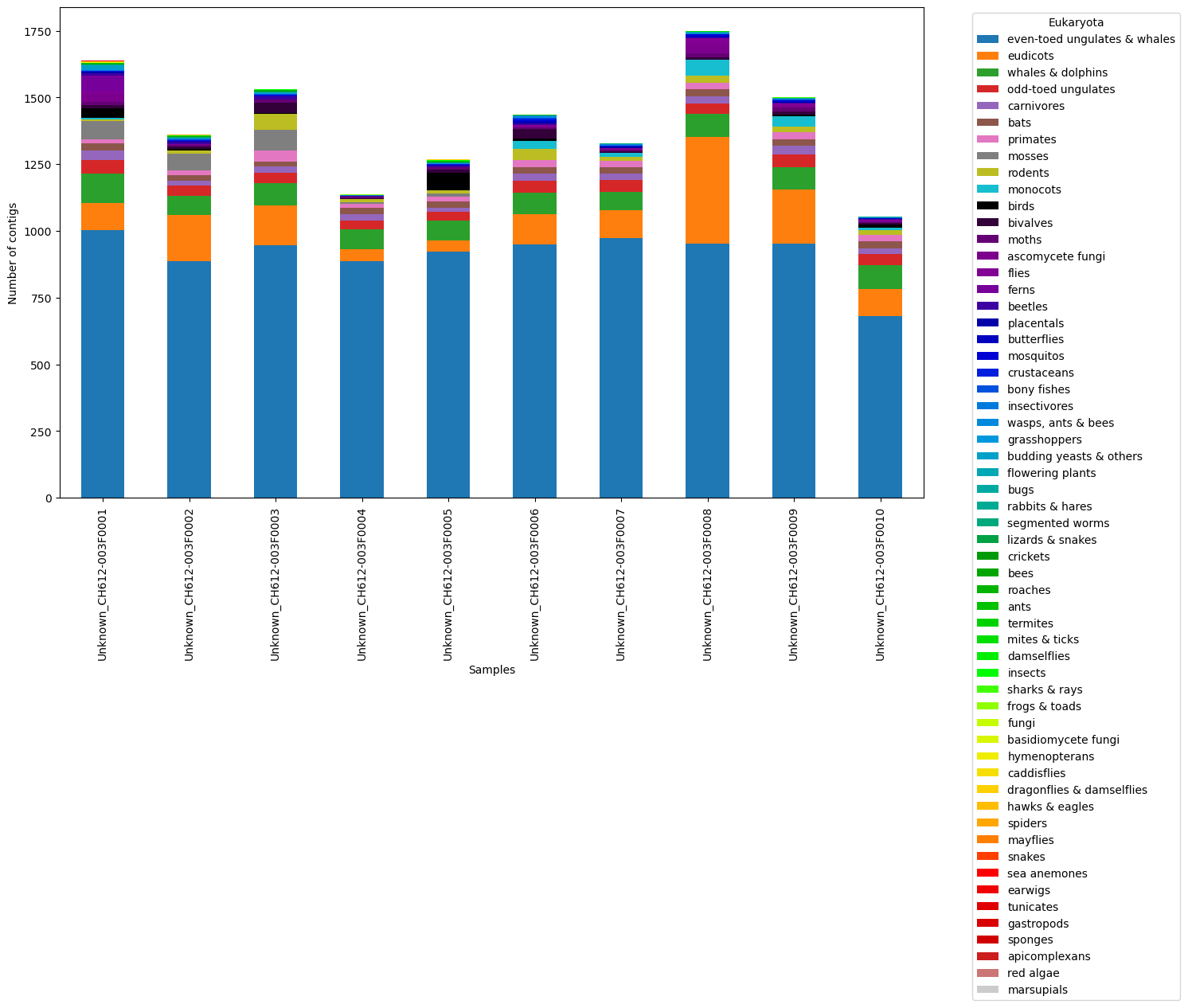

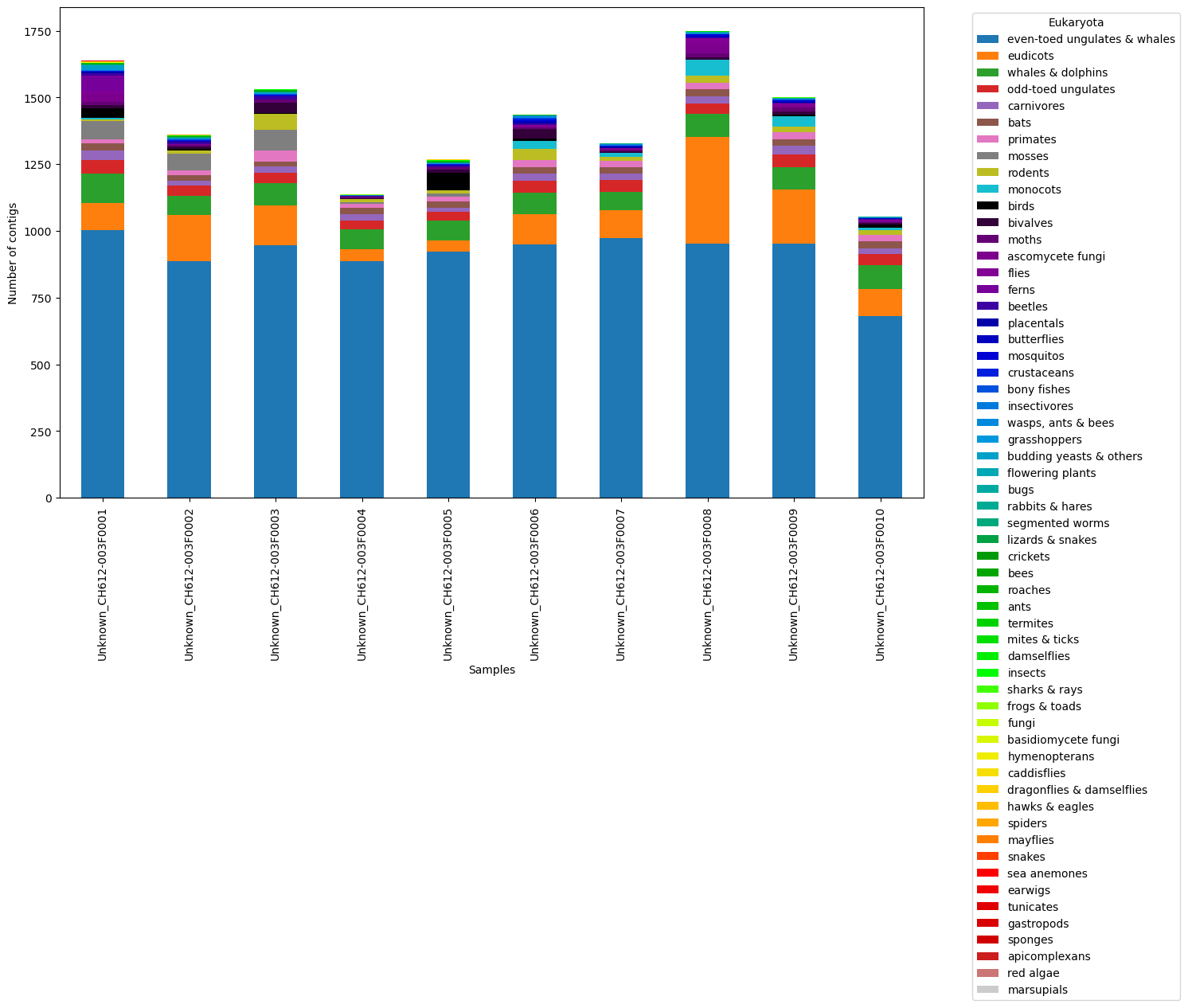


Figure S5: Number of hits with eukaryotes in pig WGS set. Contig count is given on the y-axis, sample numbers on the x-axis


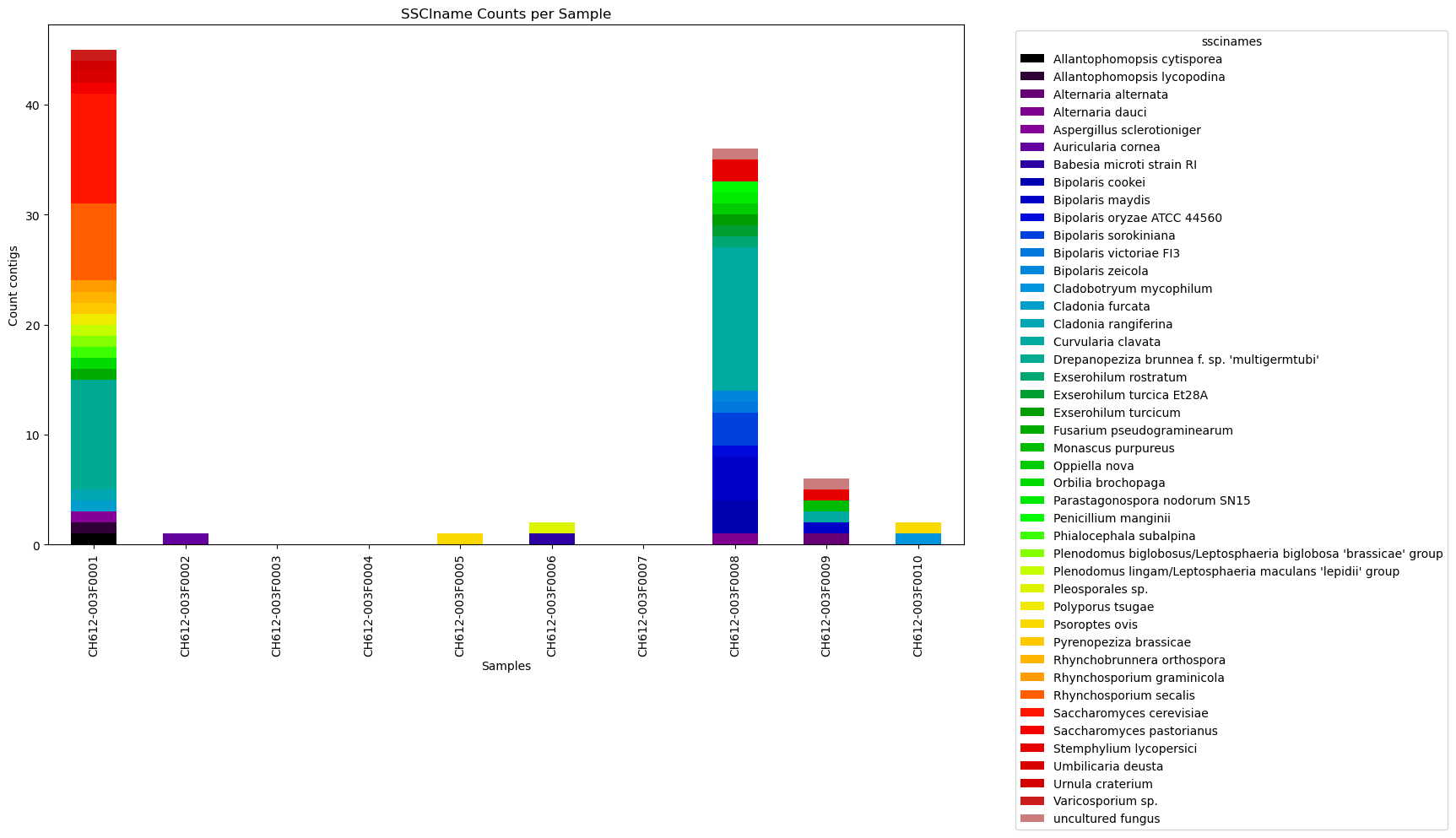

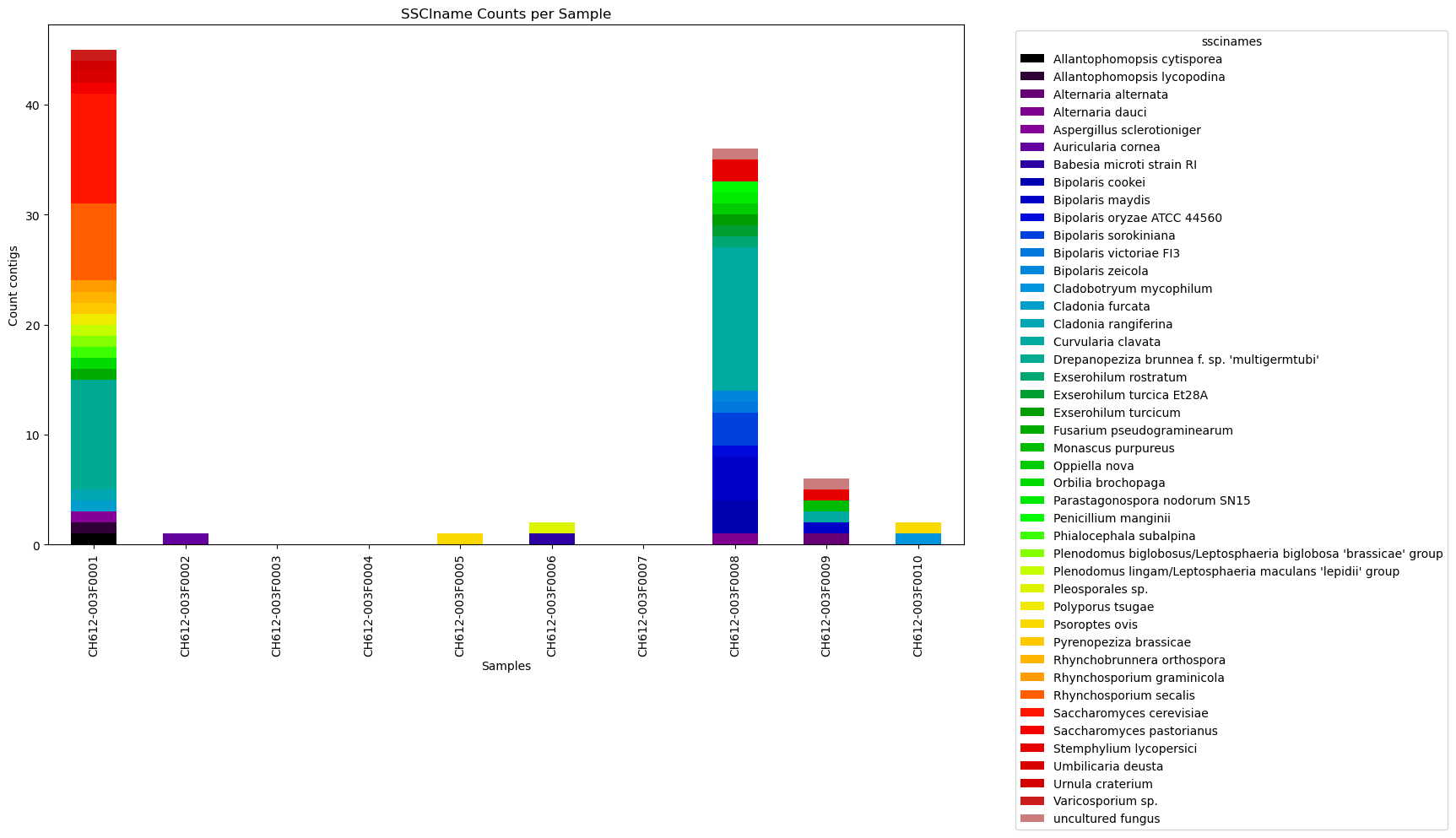


Figure S6: Number of hits with fungi, mites, and ticks in pig WGS set. Contig count is given on the y-axis, sample numbers on the x-axis

**
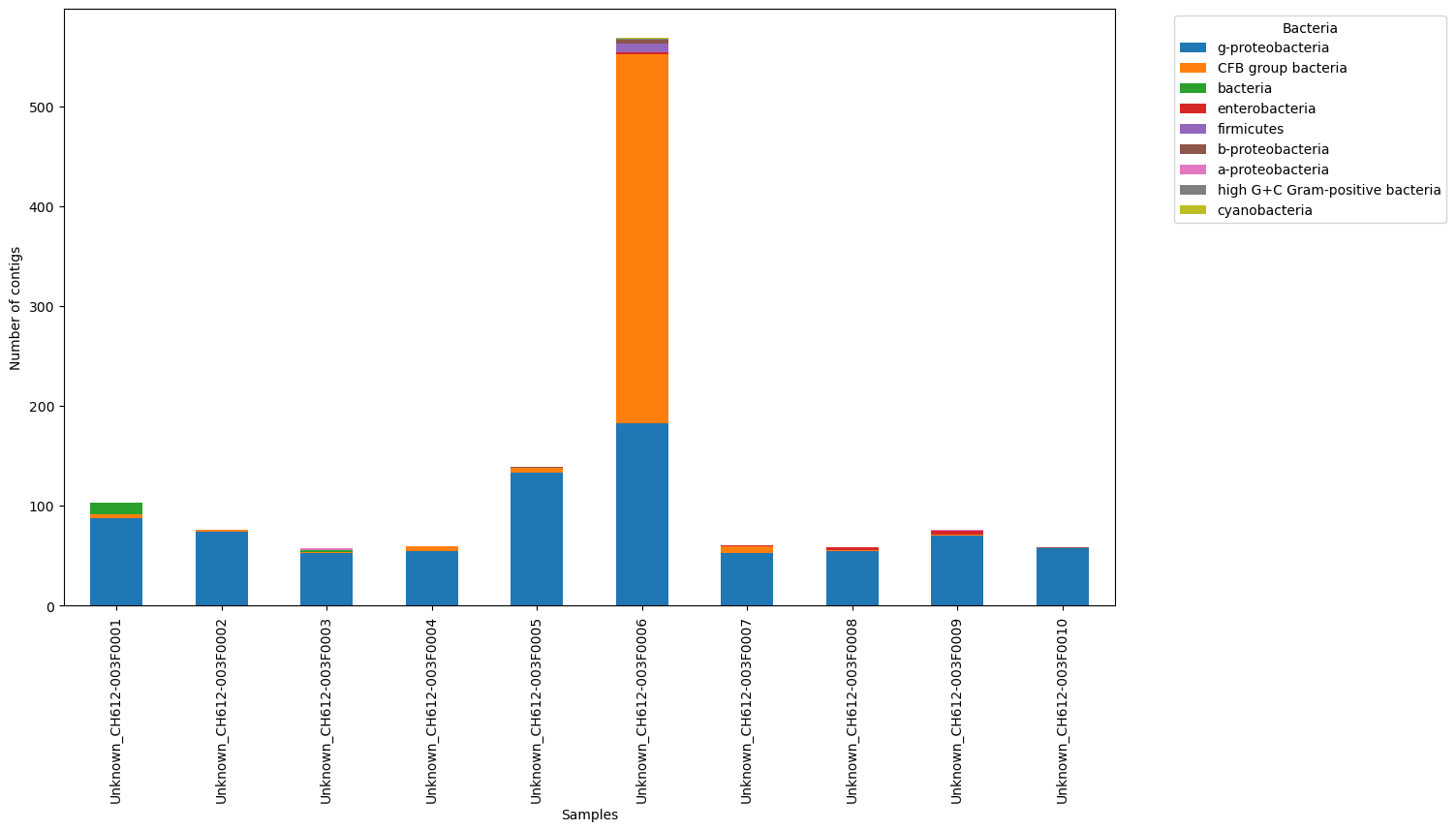
**

Figure S7: Number of hits with bacteria in pig WGS set. Contig count is given on the y-axis, sample numbers on the x-axis


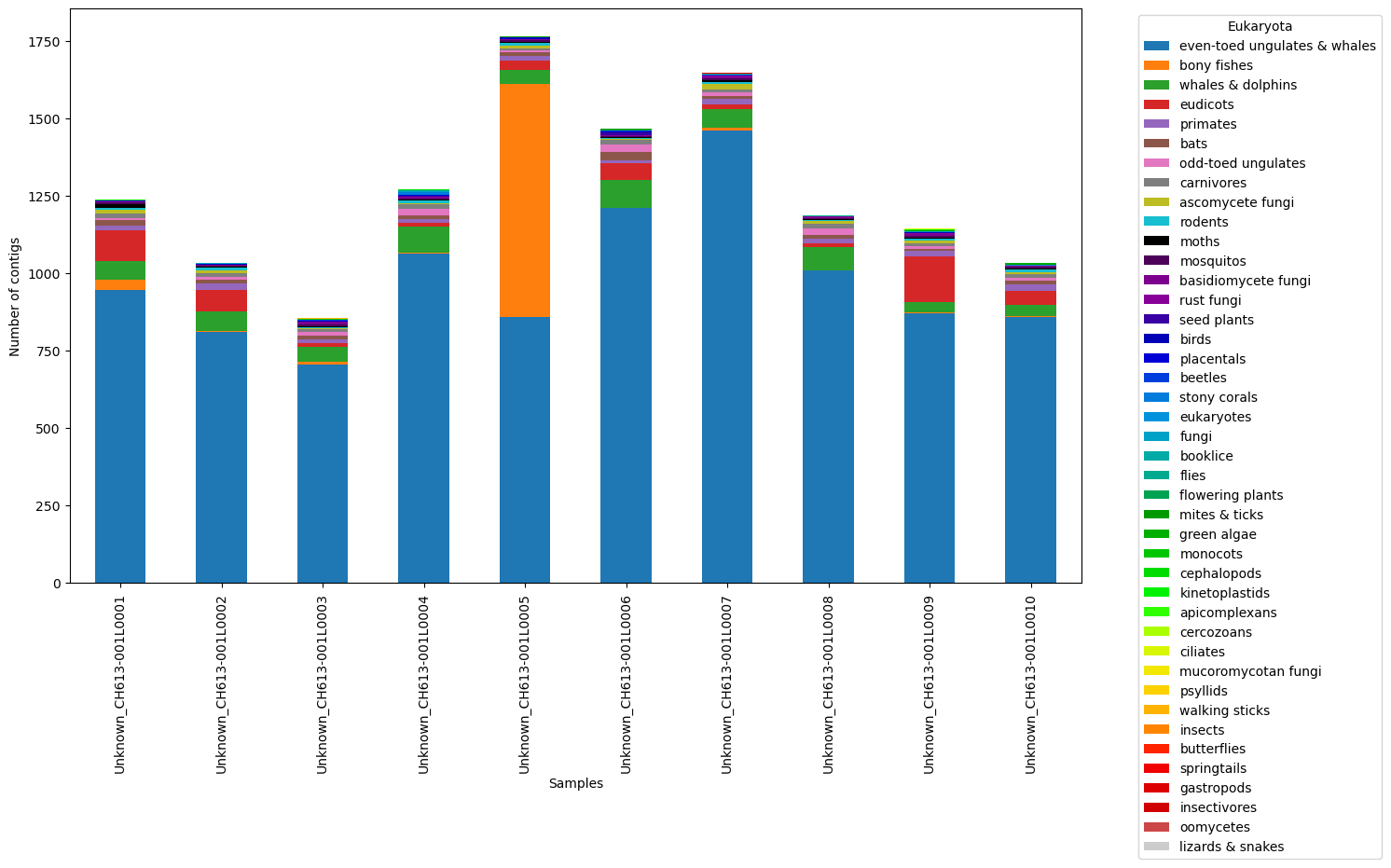


Figure S8: Number of hits with eukaryota in pig RNA-seq dataset. Contig count is given on the y-axis, sample numbers on the x-axis


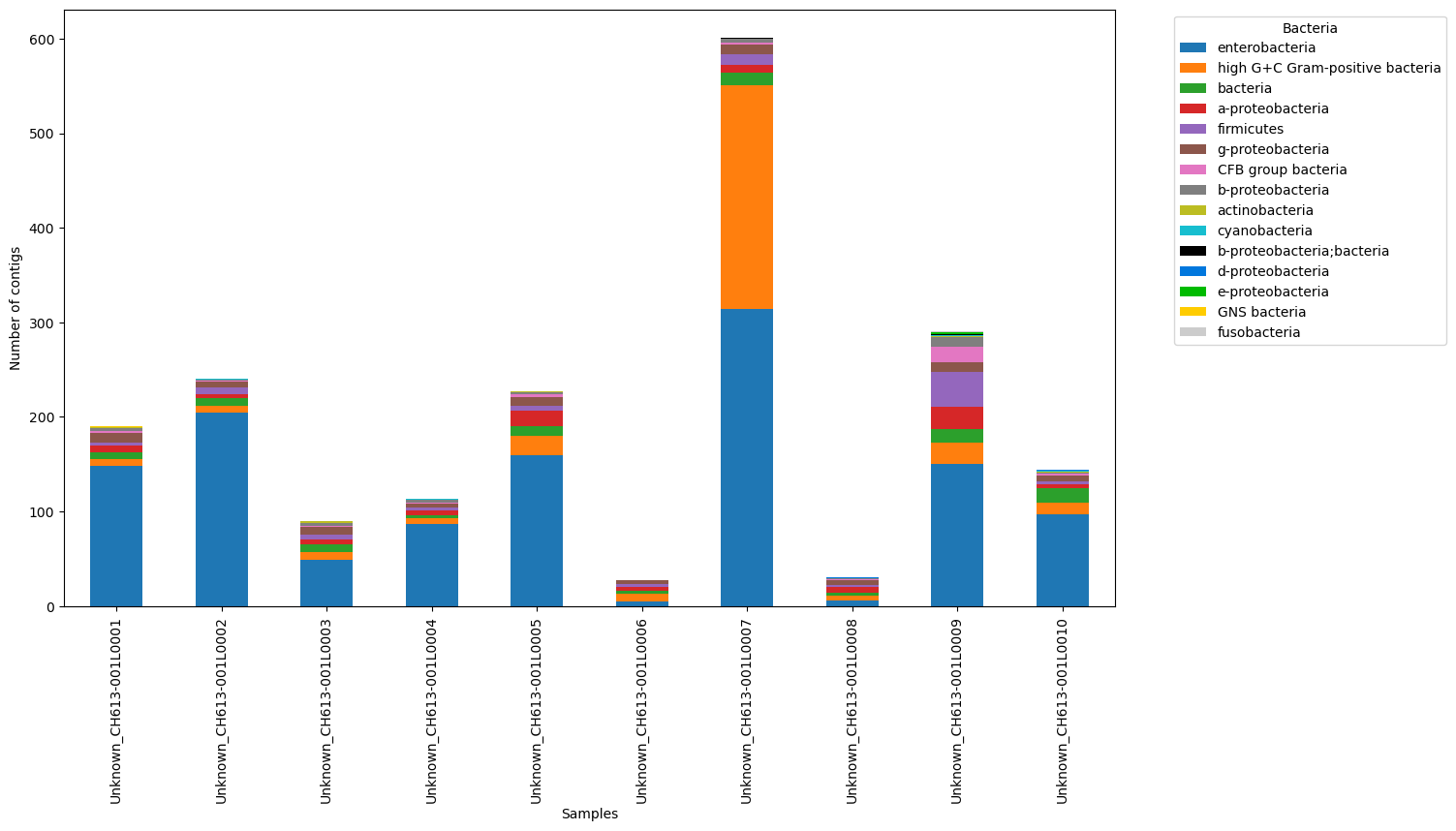


Figure S9: Number of hits with bacteria in pig RNA-seq dataset. Contig count is given on the y-axis, sample numbers on the x-axis.
